# The use of quality assurance instruments and methods to integrate diversity aspects into health professions study programmes

**DOI:** 10.15694/mep.2018.0000053.1

**Published:** 2018-03-06

**Authors:** Sabine Ludwig, Yadira Roa Romero, Johanna Balz, Mandy Petzold

**Affiliations:** 1 Charité - Universitätsmedizin Berlin

**Keywords:** Diversity, Gender, Quality assurance, Evaluation, Accreditation

## Abstract

This article was migrated. The article was marked as recommended.

Background

With the increasing diversity in our population, future medical doctors need to have adequate diversity and gender competencies in order to provide adequate and good quality of medical care. Diversity, especially sex and gender aspects, were therefore systematically integrated into the new modular medical curriculum at Charité - Universitätsmedizin Berlin. The aim was to integrate diversity aspects into further study programmes of the Charité Berlin by assessing the current degree of integration and the relevance for the professional work of students and graduates and identifying suitable and effective quality assurance instruments.

Methods

After the curriculum development of the new modular medical curriculum was completed, the gender and diversity change agent was transferred from the curriculum development team to the quality assurance section of the Office of the Dean of Student Affairs. The change agent identified in cooperation with the quality assurance team the accreditation process, student evaluations and graduate surveys as suitable methods for the integration. Furthermore, the change agent provided support to the programme directors and coordinators with the integration. The impact of the measures and instruments used for the integration are measured within the reaccredition process of the programmes.

Results

Diversity aspects could be integrated into the accreditation standards admission, curricular structure, didactics, assessment and student counselling. In the student evaluations and graduate surveys gender and diversity items like migration background, number of children, caring responsibilities, disabilities and economic status could be integrated. Furthermore, students and graduates were asked to evaluate the relevance of gender and diversity competencies for their professional work and the degree of the curricular integration.

Discussion and Conclusion

The impact of the integrated diversity aspects can only be evaluated within the accreditation process. In order to increase and improve the awareness of students and faculty members of diversity aspects and issues the support by a gender and diversity change agent with the integration and by lecturing on this subject is important. A gender and diversity sensitive accreditation process contributes to the reflexivity and awareness of the faculty members involved.

## Introduction

The increasing diversity in our population as well as in the student body and the patients makes it necessary that gender and diversity aspects are adequately integrated into medical education as well as in other health professions education ( Dogra, Giordano, & France, 2007; Horval, Horey, Romios, & Kis-Rigo, 2014). This contributes to an improved quality of medical care for different patient and social groups ( Ahmed, 2012; Awosogba et al., 2013; Verdonk & Abma, 2013). In health professions education, diversity aspects are relevant as curricular content and learning objectives ( *fixing the content*) ( Verdonk & Janczukowicz, 2018), diversity sensitive didactics, diversity sensitive institutional framework as well as a diversity sensitive study environment ( *fixing the institution*) and admission processes ( *fixing the numbers*) ( Kai, Spencer, & Woodward, 2001).

Future health professionals therefore need to have adequate knowledge on relevant diversity aspects in diseases, especially sex and gender differences in the diagnosis, pathogenesis, manifestation, therapy and prevention of diseases ( Regitz-Zagrosek, 2012; Weiss & Levison, 2000;). They need to be able to reflect their own gender role, be diversity sensitive in their communication skills ( Hall et al., 1994), take diversity, especially sex and gender aspects, into account when designing, conducting and interpreting their study results ( Clayton & Collins, 2014; CPME, 2016). Diversity sensitive didactic methods imply the use of gender and diversity sensitive language in the courses and the teaching materials, the integration of gender and diversity aspects into case studies and the awareness and knowledge on culturally based differences in learning behavior and study progress and adequate methods to work with this ( Derichs-Kunstmann, 2000). For the study environment it is important, that family friendly study timetables are provided, that courses are hold in small groups and that course facilities are accessible for students with mobility impairment. In the admissions processes students are not discriminated because of their age, sex/gender, disabilities, family caring tasks or ethnic background ( Ebenfeld, 2017).

There have been efforts to integrate diversity, especially sex and gender aspects, into the new undergraduate modular medical curriculum at Charité ( Hitzblech, Maaz, & Peters, 2014; Maaz et al., 2018) as teaching content and learning objectives. For this a gender and diversity change agent was placed into the curricular development team and developed a ten step approach as well as an institutional framework for the integration. With this approach 223 (5%) learning objectives, 94 (21%) lectures, 33 (12%) seminars and 16 (8%) practical courses on diversity issues, especially sex and gender aspects, could be integrated ( Ludwig et al., 2015). Based on the actual evaluation standards ( Ludwig et al. 2015; Verdonk, Mans, & Lagro-Janssen, 2005;) the integration was successful. However, the integration into the medical curricula of other German medical faculties has been slow and not systematic as a survey by the German Medical Women’s Association has shown. Only one faculty has achieved a full integration based on the actual standards. On a national level further integration is therefore needed also in the curricula of other health professions ( Ludwig, Dettmer, Peters, & Kaczmarczyk, 2016).

Although, there have been measures to integrate gender and diversity issues, the evaluation of students of all semesters and all study programmes at Charité (N=1150), that was conducted in 2015, has shown that 24,7% of students have experienced discrimination during their studies (Bereich Qualitätssicherung, 2015). This results shows the need for more gender and diversity awareness and sensitivity in the faculty and the integration of diversity issues into all health professions curricula.

Here, the aim was to integrate diversity, especially sex and gender aspects, into the other health professions study programmes of Charité. For this, information on the relevance and the degree of integration of diversity issues was necessary as well as the identification and development of suitable and effective quality assurance instruments and tools for the integration.

## Methods

After the curricular planning of the last module of the new undergraduate medical programme, the gender and diversity change agent ( Ludwig et al. 2015) was transferred from the curriculum development team to the quality assurance section with the task to establish methods for the integration of gender and diversity aspects into the other study programmes of Charité and identify and develop adequate quality assurance instruments and tools for the integration.

For the integration of diversity issues, the six categories for diversity from the General Equality Treatment Act ( AGG, 2006) were used: gender/sex, age, ethnicity, disabilities, sexual orientation and religion. Besides those categories, aspects like social and economic status, education, colour of hair or skin and body shape were also taken into consideration.

Among the different quality management instruments, the gender and diversity change agent identified the internal accreditation process (1), student evaluations and graduate surveys (2) and course evaluations (3) as suitable for the integration as well as giving support and advice to the programme directors and coordinators (4).

(1) In Germany, there are two approaches to accreditation. A faculty receives system accreditation if the quality insurance system of the university or faculty is accredited. The second approach is the *accreditation* of degree programmes (programme *accreditation*). As the first medical faculty in Germany, the Charité - Universitätsmedizin Berlin received system accreditation in 2015. Internal accreditation of degree programmes is therefore coordinated by the quality assurance section. The change agent developed standards and criteria for diversity and gender sensitive accreditation in close cooperation with the quality assurance team ( Schlüter, Kortendiek, Hilgemann, & Knauf, 2012).

(2;3) In the evaluation of students in their last year of studies and the graduate surveys students and graduates are asked, besides the sociodemographic items, to evaluate the study environment, curricular contents, structures, the quality of teaching, assessment tools, preparedness for their future professional work, the relevance and degree of curricular integration of different competencies, career perspectives and plans. In the graduate surveys items on career entry and the current employment situation are also included. The change agent developed diversity and gender sensitive items for the evaluation tools including the course evaluations. For the migration background the item developed by Schenk et al. ( Schenk et al., 2006) was used. The disability item is a standardized item being developed by the International Center for Higher Education Research for graduate surveys ( INCHER, 2014) Furthermore, items were integrated to evaluate the relevance of gender and diversity competencies for their future professional work and the degree of the integration of those issues.

(4) The change agent developed a diversity training for students in their final years and offered support and provided counselling to the programme directors and coordinators for the integration of diversity aspects. Close cooperation with the equal opportunities officer and the office for family affairs was necessary to develop measures to support vulnerable students groups.

## Results

The gender and diversity change agent managed to integrate gender and diversity aspects into the identified quality assurance tools in close cooperation with the quality assurance team.

### Accreditation

The gender and diversity change agent followed the accreditation of the study programmes Master of Health Professions Education, Master of Molecular Medicine, Master of Public Health, Master of International Health and Master of Medical Neurosciences. The accreditation of the Master of Health Professions Education and International Health were accreditations with on-site visits. The accreditation of the study programme Master of Molecular Medicine, Master of Public Health and Master of Medical Neurosciences were concept accreditations. For the accreditation of degree programmes external experts and reviewers are invited. For concept accreditations a minimum of three experts is required and for accreditation with on-site visits a minimum of four experts are required. The gender and diversity change agent checked for the equal gender representation of the experts. For the accreditation of the Master of Health Professions Education there were four female experts, for the Master of Molecular Medicine two male and one female, for the Master of Public Health two male and one female, for the International Health accreditation three female and one male expert and for the Master of Medical Neurosciences two female and one male.

Furthermore, the programme coordinators need to prepare a detailed report for the accreditation process, explaining the status quo of the achievement of the different standards and criteria. The gender and diversity change agent managed to integrate gender and diversity criteria for the following accreditation standards in close consultation with the quality assurance team:

#### Admission process

In the standard for the admission process the criteria *“students are not being discriminated against based on their sex, gender, age, disabilities, migration background or family caring tasks”* could be integrated.

#### Structure of the study programme and qualification goals

In the standard of the structure of the study programme and qualification goals the gender and diversity criteria “ *diversity and gender differences are integrated into the teaching content and are part of the curriculum*” could be integrated.

#### Assessment

In the standard for the assessment the criteria “ *student are not being discriminated against based on their age, sex and gender, disabilities, migration background or family caring tasks.*” This implies equal opportunities for all students.

#### Student Support and Counselling

In the standard student support and counselling the criteria *“support and counselling is offered to students with family tasks and disabilities”* could be integrated.

#### Gender equality, equal opportunities and diversity

Before, this standard focused only on gender equality and equal opportunities. The change agent added “diversity”. Furthermore, the following criteria could be included: “ *the study programme collects data of the study progress of foreign students, students with disabilities and students with migration background. Those data also include the development of the gender ratio and age of the students*” as well as the criteria *“the organization of the study programme takes the need of students and teachers with family tasks and disabilities into account”* and *“gender-sensitive didactic methods are used.”*

The integration of those criteria into the accreditation process already had some impact and led to the development of further measures to support students with children, e.g. the faculty finances the position of a student counselling and giving advice to students with children. Furthermore, in the experts and evaluators report of the Master of International Health study programme the programme directors are asked to encourage and support the application of students with disabilities (suggestion 5; expert and evaluators report 2017.)

### Student evaluations and graduate surveys

Gender and diversity sensitive items could be integrated into the different student evaluations and graduate surveys. They were integrated into the student evaluations after the first semester, the student evaluations during the last year of studies and the graduate surveys and evaluations of the Bachelor of Health Sciences, Master of Health Professions Education and the medical study programmes. Items of different sexual orientation besides male, female, „others“ could be integrated as well as age, the number of children, caring responsibilies, disabilities, migration background, family status, economic status and educational background of partner.

#### Children and family care tasks

43% of the graduates of the Bachelor of Health Sciences (2016; N=83; response rate 39%; N=32) have children and 50 % have child care tasks, 35,7% mainly take care of the children themselves and 10,7% the partner is responsible for child care, for 7,1% the childcare is equally distributed between the partners. 25% of the graduates of the Master of Health Professions Education (July 2017; N=30, response rate 40%; N=12 have children), 8,3% share the childcare tasks with the partner. The evaluation of the medical students in their last year of studies (N=886; response rate 22,6%; N=200) has shown that 9,5% have child caring tasks and 2% have other family care tasks.

#### Migration Background / Nationality

96,9% of the graduates of the Bachelor of Health Sciences are German, only one has a nationality from a different EU member state. All graduates have German as their native language. All graduates of the Master of Health Professions Education study programme have German nationality and German as native language, whereas the evaluation of the medical students in their last year of studies has shown that 20,7% have migration background.

#### Disabilities

The graduate survey of the Bachelor of Health Sciences (N=83; response rate 39%; N=32) has shown that 12,5% of the students who participated in the survey experience psychological disorders (depression, eating disorders, addiction, psychosis), 9,4% are students with mobility impairment and 15,6% have chronic somatic disorders like Asthma, Diabetes, gastrointestinal diseases or Rheumatoid Arthritis.

16,7% of the graduates of the Masterprogramme Health Professions Education have psychological disorders (depression, eating disorders, addiction, psychosis), 16,7% are students with mobility impairment, 8,3% of students have visual impairment.

The survey of students in their final year of medical studies has shown that 14,4% of the students experience psychological disorders (depression, eating disorders, addiction, psychosis), 9,4% chronic somatic disorders, 3,5% mobility impairment, 2,5% visual impairment and 1% hearing impairment.

#### Relevance and degree of curricular integration of gender and diversity competencies

Students and graduates are asked to evaluate the importance of gender and diversity competencies into the curriculum. 45,2% of the graduates of the Bachelor of Health Sciences considered gender competencies and 50% intercultural competencies as relevant whereas half of them evaluated the degree of integration into the curriculum as minor (51,6%; 55,2%).

The graduate survey of the Master of Health professions education has shown that 54,5% considered gender-sensitive competencies, 27,2% intercultural competencies and 40% competencies to be able to deal with patients and clients with disabilities as relevant for their professional work. 82% consider the degree of integration of gender competencies, 60% the degree of integration of cultural competencies and 91% the degree of integration of competencies to deal with patients and clients with disabilities as minor.

The evaluation of students in their last year of studies (N=835; response rate 22%, N=184) has shown that students of the new modular medical curriculum and students of the traditional curriculum considered gender competencies as relevant for their future work as physician (62%; 63%) as well as cultural competencies (76%;71%). In contrast, 69% of students of the traditional curriculum evaluated the degree of integration of gender competencies as minor, while 83% of students of the new programme rated the degree of integration as extensive.

### Course evaluations

The gender and diversity sensitive items *“The teacher was gender sensitive and has taken the diversity of the student body into account”* and *“the interaction of students and teacher(s) was friendly and respectful”* could be integrated into the course evaluations in close cooperation with the quality assurance team.

### Development of a course for diversity competencies

The change agent developed a course for students in the final year of the Master of Health Professions Education. 74% of the students evaluated the course as useful for their future professional work.

## Discussion and Conclusion

Integration of diversity, especially gender and sex aspects, has made some progress. There have been efforts in several European countries, for example at Radboud University in the Netherlands ( Lagro-Janssen, 2010), Umea University and the Karolinska Institute in Sweden ( Andersson, Salander, & Hamberg 2013) as well as several universities in Austria ( Hochleitner, Nachtschatt, & Siller, 2013). On a European level, the Standing Committee of European Doctors has published a position paper emphasizing the importance of the integration of gender and sex aspects into medical education (CPME, 2016). Also in Canadian ( Zelek, Phillips, & Lefebvre, 1997) and American medical schools ( McGregor, Templeton, Kleinman & Jenkins, 2013; Miller et al., 2013; Schiebinger, 2008) sex and gender sensitive curricula are being developed. Standards for a successful integration have been defined by Verdonk et al. ( Verdonk et al., 2005). Ludwig et al. have developed a ten step approach for the integration ( Ludwig et al., 2015). A survey by the German Medical Women’s Association has shown that only one faculty has achieved full integration of gender and sex aspects based on the current evaluation standards and only two faculties have a change agent ( Ludwig et al., 2016). Muntinga et al. have mapped a medical curriculum in the Netherlands on the inclusion of diversity and found out that although there was a supportive climate, the diversity-responsiveness of teaching material is yet to be improved (Muntinga, Krajenbrink, Peerdemann, Croiset, & Verdonk, 2015). The working group on gender and diversity in medical education of the German Association for Medical Education has proposed 207 gender and diversity learning objectives for the new national competency based learning objectives catalogue in medicine. 82 gender and diversity learning objectives were integrated, 4% of all learning objectives. This catalogue can provide guidance for the curricular integration (NKLM, 2017).

With the transfer of the change agent from the curriculum development team to the quality assurance section adequate quality assurance tools and instruments for the integration of diversity and gender aspects were identified and gender and diversity sensitive criteria and items developed. Gender and diversity issues could be integrated into the different standards and criteria of the accreditation process of several health professions study programmes like admission process, curricular structure, assessment and student support and counselling. They could also be integrated into student evaluations and surveys as well as course evaluations in close collaboration with the quality assurance team. Furthermore, a course on diversity issues could be developed. Student surveys have shown that almost 10% of students have children and that there is a high level of students with psychological disorders. Measures to support them are yet to be developed and improved.

The graduate survey of the Bachelor of Health Sciences of 2016 and the Master of Health Professions Education 2017 have shown that students consider the integration of diversity and gender aspects as relevant, but evaluate their integration as minor, whereas the evaluation of medical students in their last year of studies has shown that students of the traditional and the new curriculum consider those aspects as relevant, but the students of the new curriculum evaluate the integration as extensive. This result indicates that the integration into the new curriculum was successful and that the methods used were effective.

### Limitations

There are several limitations to the study. One limitation is the sustainability of the integrated standards, criteria and items after the change agent is not assigned to this section anymore. Furthermore, resources and institutional support are needed for the integration. Gender and diversity sensitive criteria could be integrated into the accreditation process, but the impact of the integration can only be evaluated during the reaccreditation process of the programmes. We can only get information on the proportion of students belonging to different vulnerable groups, but we cannot identify the individual students due to data protection regulations. This makes individual support to students difficult. Although, a third sex/gender “others” could be integrated into the sociodemographic items, the number of students is very small so that results are not representative. The course on diversity issues was only integrated into the Master of Health Professions Education study programme, it needs to be integrated into the other study programmes as well.

### Conclusion

As students and graduates consider gender and diversity competencies as relevant for their future professional work, medical schools should aim at a systematic, far-reaching curricular integration of diversity- and gender-sensitive perspectives and learning. Gender and diversity sensitive accreditation can therefore contribute to fixing the numbers by integrating it e.g. into the standards of the admission process, to fixing the institutions e.g. by family-friendly study schedules or and barrier-free accessible facilities and to fixing the content. The integration into graduate surveys, student and course evaluations helps to assess the actual diversity of students and the proportion of possible vulnerable groups in order to develop measures to support them. Furthermore, it helps to evaluate the relevance and degree of curricular integration and the gender and diversity awareness of teachers and faculty members. The placement of a gender and diversity change agent into the quality management and evaluation section and the use of quality assurance instruments for the integration has proven to be an effective approach to improve the gender and diversity awareness of students and faculty members. Further student and graduate surveys are necessary to measure the increased gender and diversity competencies of the students and graduates and their impact on the quality of medical care and patient outcomes.

## Notes On Contributors

Dr. Sabine Ludwig has obtained her doctorate degree from the Charité - Unversitätsmedizin berlin in the field of Women’s Health, Gender Medicine and Curriculum Development. She used to work in the quality assurance section of the Charité Berlin. Currently, she is working at the Robert Koch Institute where she is in charge of the women’s health report for Germany.

Dr. Yadira Roa Romero is a psychologist with a PhD in neuroscience. She currently works as a research associate and member of the quality assurance team at the Charité - Universitätsmedizin Berlin. Her research focuses on student surveys in medical education and public health.

Johanna Balz is a psychologist. She is currently working on her PhD in the field of clinical neuroscience. Johanna was working as a research associate in the quality assurance section at the Charité - Universitätsmedizin Berlin and is currently focusing on coordination issues and research in medical education.

Mandy Petzold holds an an advanced degree in educational science. She is a quality manager and auditor as well as EFQM-Assessor. She was mainly in charge of the system accreditation of the Charité - Universitätsmedizin Berlin which was successfully concluded in 2015. Currently, she leads the multiprofessional team of quality assurance at Charité - Universitätsmedizin Berlin.
