# Supplementary material for: The use of quality assurance instruments and methods to integrate diversity aspects into health professions study programmes
Source: MedEdPublish (2016). 2018 Mar 6;7:53. [Version 1] doi: 10.15694/mep.2018.0000053.1 (PMC10711966; doi:10.15694/mep.2018.0000053.1)
Supplement: Peer review history

# Reviewer report: Reviewer response for version 1

**DOI:** 10.21956/mep.19519.r28882

**Published:** 2018-03-26

**Authors:** Janusz Janczukowicz

**Affiliations:** 1 Medical University of Lodz

This review has been migrated. The reviewer awarded 4 stars out of 5 This is really good to see a paper focusing on integrating gender and other diversity dimensions into quality assurance in health professions education not only form the perspective of curriculum content but also the educational climate and inclusiveness, comprising students’ perception of their teacher’s attitudes towards diversity. I hope that this holistic approach to quality assurance will be further developed and implemented by the other universities. I would be happy to read why the phrase “gender-and-diversity” (e.g. gender and diversity competencies) is used, sort of contrasting both elements instead of presenting gender as one of elements of diversity.

Reviewer Expertise:

NA

No decision status is available

# Reviewer report: Reviewer response for version 1

**DOI:** 10.21956/mep.19519.r28884

**Published:** 2018-03-12

**Authors:** Peter Dieter

**Affiliations:** 1 AMSE

This review has been migrated. The reviewer awarded 4 stars out of 5 The term diversity is defined differently internationally (only / or): gender, ethnic group, disabled-healthy, etc. This must be considered when reading articles that relate to diversity.I enjoyed reading this well-written paper.

Reviewer Expertise:

NA

No decision status is available

# Reviewer report: Reviewer response for version 1

**DOI:** 10.21956/mep.19519.r28883

**Published:** 2018-03-06

**Authors:** Petra Verdonk

**Affiliations:** 1 VU University Medical Center, dpt Medical Humanities

This review has been migrated. The reviewer awarded 4 stars out of 5 Earlier at Charité in Berlin, a large scale gender mainstreaming project has been conducted in medical education. Currently, a next step is taken to mainstream gender and diversity in five Master programmes including international health and public health programmes. In this study, the authors look beyond curriculum content (fixing the knowledge) to fixing the institutions, namely the structural embedding of gender and diversity in internal quality assurance instruments by the development of tools for integration. Additional criteria were developed for instance for the accreditation standards admission process, the representation of men and women at committees was assessed, and items were developed for course evaluations and graduate surveys such as migration background or caring responsibilities of respondents. Students and graduates were asked to evaluate the relevance of gender and diversity competencies for their professional work, and how they perceived curricular integration of diversity. This enterprise, as well as research on how quality assessment can be improved is highly important. Surely, when diversity is not structurally embedded, we run the risk of diversity outstreaming at the next curriculum reform, a change in course coordinator, or a new education policy at university level. I enjoyed reading this well-written paper, as the authors really hit home with their broad approach of diversity in quality assessment and further inclusive education at all level. At the level of students’ perceptions of ‘worthwhile knowledge’ some work still seems necessary, and the dependency of a change agent still warrants attention, but when it comes to pushing the matter forward, Charité, again, is ahead of the rest of us.

Reviewer Expertise:

NA

No decision status is available
